# Toll-like receptors and COVID-19: a two-faced story with an exciting ending

**DOI:** 10.2144/fsoa-2020-0091

**Published:** 2020-07-30

**Authors:** Livia Onofrio, Michele Caraglia, Gaetano Facchini, Vincenzo Margherita, Sabino De Placido, Carlo Buonerba

**Affiliations:** 1 Department of Clinical Medicine & Surgery, University of Naples “Federico II”, Naples, Italy; 2 Department of Precision Medicine, University of Campania “L. Vanvitelli”, Naples, Italy; 3 Medical Oncology, ASL Napoli 2 Nord, “S.M. delle Grazie Hospital”, Pozzuoli (NA), Italy; 4 Regional Reference Center for Rare Tumors, Department of Oncology & Hematology, AOU Federico II of Naples, Naples, Italy; 5 National Reference Center for Environmental Health, Zoo-prophylactic Institute of Southern Italy, Portici, Italy

Coronavirus disease 19 (COVID-19) is caused by a novel single-stranded RNA beta-coronavirus called severe acute respiratory syndrome coronavirus 2 (SARS-CoV-2) [1]. COVID-19 has quickly become a pandemic since the first case was diagnosed in Wuhan, China, in December 2019, with currently over 5 million people infected and over 340,000 deaths in more than 200 different countries [2]. SARS-CoV-2 molecular structure presents four major proteins named the spike (S), envelope (E), membrane (M) and nucleocapsid (N). Approximately 80% of SARS-CoV-2 genomic sequence is in common with SARS-CoV, the virus responsible for the SARS outbreak in 2002 [3]. Despite close similarities, the SARS-CoV-2 Spike protein – which allows the virus to bind to the ACE2 receptor – is several amino acids longer than the SARS-CoV Spike protein, which may represent the key reason why COVID-19 has spread so rapidly throughout the world, inherently different from SARS, which was quickly contained [3].

From a clinical point of view, SARS-CoV-2 infection is highly heterogeneous. In a report including over 40 thousand cases diagnosed in China [4], mild disease was reported in approximately 80% of patients, severe signs or symptoms including dyspnea, hypoxia or lung infiltrates involving >50% of the parenchyma occurred in 14% of patients, while signs indicative of critical disease such as shock, respiratory failure or multiorgan dysfunction were reported in 5% of cases. Of note, mortality was 2.3% in the entire cohort. With such a highly heterogeneous clinical course, SARS-CoV-2 infection poses a challenge for researchers to define its underlying biological mechanisms as well as for clinicians to establish the optimal therapeutic approach, which remains elusive at the present time.

While most patients infected with SARS-CoV-2 remain asymptomatic or show mild disease, the clinical course in those who develop severe COVID-19 includes onset of dyspnea after 5–6 days, necessity of hospitalization after 7–8 days and development of acute respiratory distress syndrome (ARDS) after approximately 8–12 days from onset of symptoms [5]. Mortality in patients admitted to the intensive care unit can be as high as 60% [6]. Current therapeutic management of patients with severe COVID-19 is primarily based on ventilation support, with a potential role played by a few pharmacological agents. Remdesivir, a nucleotide analog with *in vitro* evidence of anti-SARS-CoV-2 activity, has shown encouraging activity against severe COVID-19. Remdesivir versus placebo was associated with an improvement in time to recovery in a preliminary report involving 1063 randomized COVID-19 patients with severe respiratory disease, although mortality remained high in both arms (mortality at 14 days: 7.1 vs 11.9% with remdesivir vs placebo, hazard ratio for death, 0.70; 95% CI: 0.47–1.04) [7]. Another retrospective study reported about 20 patients with severe COVID-19 treated with tocilizumab, a monoclonal antibody directed against IL-6, a key player in the so-called ‘cytokine storm’ associated with COVID-19 ARDS. With 15 patients being able to decrease their oxygen intake, one being able to breath in ambient air, and no reported deaths, tocilizumab demonstrated some efficacy [8]. Results from a large prospective trial with tocilizumab [9] are pending. Although other pharmacological agents have been proposed or tested, including azithromycin, recombinant soluble ACE2, lopinavir/ritonavir [10] as well as eculizumab [11], effective pharmacological agents against such a deadly disease remain a compelling need at the present time.

Toll-like receptors (TLRs) [12] may be involved both in the initial failure of viral clearance and in the subsequent development of the deadly clinical manifestations of severe COVID-19 – essentially ARDS with fatal respiratory failure. TLRs are ubiquitously present in the animal kingdom. In humans, the TLR family comprises ten members (TLR1–TLR10), which are expressed in innate immune cells such as macrophages as well as in epithelial and fibroblast cells. Activation of TLRs can be induced by a multitude of pathogen-associated molecular patterns (PAMPs) present in bacteria, viruses and other foreign organisms. TLRs play a major role in the initiation of innate immune responses, with the production of inflammatory cytokines, type I IFN and other mediators. TLRs can be localized either on the cell surface, such as TLR-1, -2, -4, -5, -6, -10 or in the endosome compartment, such as TLR-3, -7, -8, -9 [12]. Importantly, while TLR3 recognizes viral double-stranded RNA (dsRNA), TLR7 recognizes viral single-stranded RNA and is therefore, likely to be implicated in clearance of SARS-CoV-2 [13]. TLR activation via MyD88-dependent and TRIF-dependent pathways causes nuclear translocation of the transcription factors NF-κB, IRF-3 and IRF-7, with production of innate pro-inflammatory cytokines (IL-1, IL-6, TNF-α) and type I IFN-α/β, which are essential for anti-viral responses [13]. Similarly to SARS-CoV, SARS-CoV-2 may prevent a successful immune response in infected individuals who progress to severe COVID-19 via inhibition of the TNF-receptor-associated factors (TRAF) -3 and -6, which play an essential role in inducing IRF-3/7 in response to TLR-7 activation. Available agonists against TLR-7 may prevent onset of severe COVID-19 in symptomatic patients and synergize with active anti-viral therapy.

In experimental mouse models of ARDS induced by multiple noxae, including SARS-CoV, genetic inactivation of the TLR-4 gene, but not of TLR-3 or 9 genes, was associated with reduced acute lung injury [14]. An improvement was also noted in IL-6-/- mice, which is consistent with the promising results obtained with tocilizumab. In patients with severe COVID-19, lung macrophages may play a key role in the massive release of IL-6 and other cytokines, including TNF-α, IL-1β, IL-10 and IL-12 via TLRs activation [15]. Of note, in an *in vitro* model, stimulation of human lung macrophages with subtype-selective agonists against various TLRs demonstrated that TLR4 stimulation induced the strongest effect in terms of cytokine release. Although SARS-CoV-2 is unlikely to activate TLR-4 directly, as TLR-4 responds to bacteria [12], one hypothesis based on a mouse model of acute lung injury is that oxidized phospholipids can be responsible for activation of TLR-4 and onset of ARDS [14]. It is interesting to note that neutrophil myeloperoxidase, which is reported to be at increased levels in COVID-19 patients, especially in those on ventilation support [16], is capable to oxidase phospholipids [17] abundant in alveolar surfactant [18]. TLR-4 may, therefore, represent a druggable target against COVID-19 via the use of TLR-4 antagonists.

Major difficulties in identifying effective therapeutic options in patients with severe COVID-19 lie in the heterogeneity of the disease and its erratic course, with some patients with mild symptoms at presentation developing sudden respiratory failure. On 1 May 2020, remdesivir was authorized by the FDA, MA, USA for treatment of severe COVID-19 requiring hospitalization, on the grounds of data obtained with the NIAID [7] and the Gilead-sponsored [19] trials. These trials enrolled hospitalized patients with different levels of respiratory insufficiency, including patients not requiring supplemental oxygen with an SpO_2_ <94% and those requiring mechanical ventilation, but it is currently unknown whether remdesivir efficacy may vary according to severity of the disease. Pending full analysis of remdesivir efficacy data, which should disclosure whether there are subgroup of patients who benefit less from treatment (e.g., those with more severe disease), we hypothesize that remdesivir may synergize with pharmacological agonists against TLR-7, which may be involved in viral escape mechanisms from immune clearance, as discussed above. In simian-human immunodeficiency virus (SHIV)-SF162P3-infected rhesus monkeys, administration of the TLR7 agonist vesatolimod during anti-retroviral therapy (ART) was associated with a delayed viral rebound after ART suspension [20]. In a Phase II trial conducted in 162 patients with hepatitis B, vesatolimod demonstrated remarkable safety in combination antiviral treatment, with signs of biological activity determined by an increase in IFN-stimulated gene mRNA expression [21]. Vesatolimod has been shown to be safe and biologically active and may be tested in COVID-19 in combination with active anti-viral therapy. Conversely, TLR-4 antagonists may be useful in patients on respiratory support with ARDS, possibly in combination with anti-IL-6 agents. Eritoran is a well-tolerated TLR4 antagonist that was tested for the treatment of severe sepsis in a large randomized controlled clinical trial, where it exhibited an excellent safety profile, although it yielded no improvement in mortality [22]. In an influenza mouse model, Eritoran was able to improve clinical symptoms and pathologic lung damage, while decreasing oxidized phospholipid and cytokine levels, as well as mortality [23].

Clinical testing of TLR agonists/antagonists may be optimized on the grounds of the following three key points. First, even patients with severe COVID-19 demonstrate a time-dependent spectrum of clinical manifestations, ranging from desaturation with no oxygen supplementation required to need of mechanical ventilation. We hypothesize that patients with less severe disease may more likely benefit from early anti-viral therapy, possibly in combination with TLR-7 agonists. Conversely, patients on mechanical ventilation suffering from ARDS may benefit from anti-IL-6 treatment, possibly in combination with TLR-4 antagonists. Second, intermediate biological markers of therapy efficacy that may also be useful in clinical practice represent a powerful tool to explore efficacy of multiple TLR-modulating agents. Signs of efficacy of TLR-7 agonists in combination with anti-viral therapy may be captured by an early drop in viral load, while decreasing IL-6 levels may capture efficacy of TLR-4 antagonists even in small patient populations. Third, the target population may include mildly symptomatic patients positive to SARS-CoV-2 with known risk factors for COVID-19 mortality, such as age and comorbidities, who may benefit from early treatment before onset of severe COVID-19.

In the current scenario in which only few therapeutic options against COVID-19 are available, targeting TLRs using pharmacological agents ready for clinical testing may provide major therapeutic advances in the fight against this deadly disease, which is unlikely to be eradicated for the next decades.
